# Supplementary material for: Identification of photoperiod-induced specific miRNAs in the adrenal glands of Sunite sheep (Ovis aries)
Source: Front Vet Sci. 2022 Jul 22;9:888207. doi: 10.3389/fvets.2022.888207 (PMC9354845; doi:10.3389/fvets.2022.888207)
Supplement: Supplementary file 1 [file Data_Sheet_1.ZIP › Supplementary file/miRDeep2 file.zip!/miRDeep2 file/Know_Structure/SPLP42A1/oar-mir-380.pdf]

miRBase precursor : oar-mir-380
Total read count : 2758
oar-miR-380-5p read count : 51
oar-miR-380-3p read count : 2704
remaining reads : 3

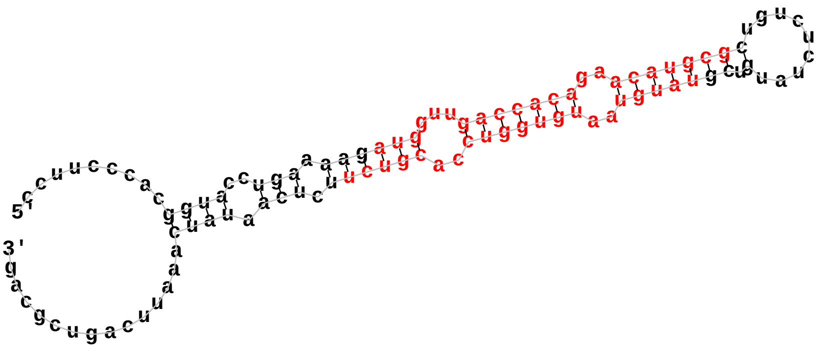

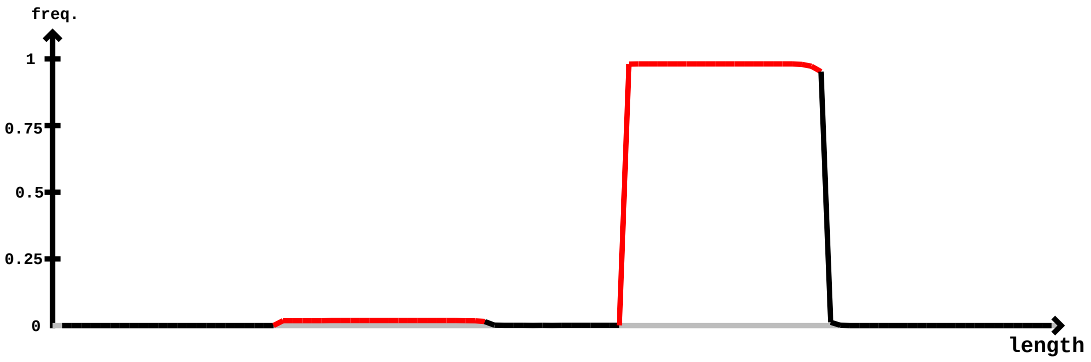

oar-miR-380-5p

oar-miR-380-3p

```
5'- ccuucccacgguaccugaaaagaugguugaccacagaacaugcgcugucucuaugucguauguaaugugguccacgucuucucaauaucaaauucagucgcag  -3'   exp
    .........((((..(((.((((((...(((((((..((((((((.........).)))))))..)))))))..)))))).))).)))).............     reads     mm       sample
    ......................augguugaccacagaacau..............................................................     1         0        seq
    ......................augguugaccacagaacaug.............................................................     1         0        seq
    ......................augguugaccacagaacaugc............................................................     9         0        seq
    ......................augguugaccacagaacaugcg...........................................................     37        0        seq
    ......................augguugaccacagaacaugcgc..........................................................     2         0        seq
    ......................augguugaccacagaacaugcgcugu.......................................................     1         0        seq
    ...........................ugaccacagaacaugcgcugucu.....................................................     1         0        seq
    .................................................ucuaugucguauguaaugugguccacgucu........................     1         0        seq
    ...................................................uaugucguauguaaugugguccacg...........................     1         0        seq
    ..........................................................uauguaaugugguccacg...........................     4         0        seq
    ..........................................................uauguaaugugguccacgu..........................     19        0        seq
    ..........................................................uauguaaugugguccacguc.........................     56        0        seq
    ..........................................................uauguaaugugguccacgucu........................     2589      0        seq
    ..........................................................uauguaaugugguccacgucuu.......................     31        0        seq
    ..........................................................uauguaaugugguccacgucuuc......................     3         0        seq
    ...........................................................auguaaugugguccacgucu........................     2         0        seq
```
